# Supplementary material for: Purification and molecular characterization of phospholipase, antigen 5 and hyaluronidases from the venom of the Asian hornet (Vespa velutina)
Source: PLoS One. 2020 Jan 10;15(1):e0225672. doi: 10.1371/journal.pone.0225672 (PMC6953831; doi:10.1371/journal.pone.0225672)
Supplement: S1 Fig — (PDF) [file pone.0225672.s001.pdf]

Images from original were captured with the software "IMAGE LAB 6.0" (BIORAD), in all cases.

In all cases, **X** corresponds to unrelated samples with what is presented in the manuscript

In all cases, the NOVEX Tricine 10-20% acrylamide were silver-stained.

SDSPAGE used in left panel of Fig1

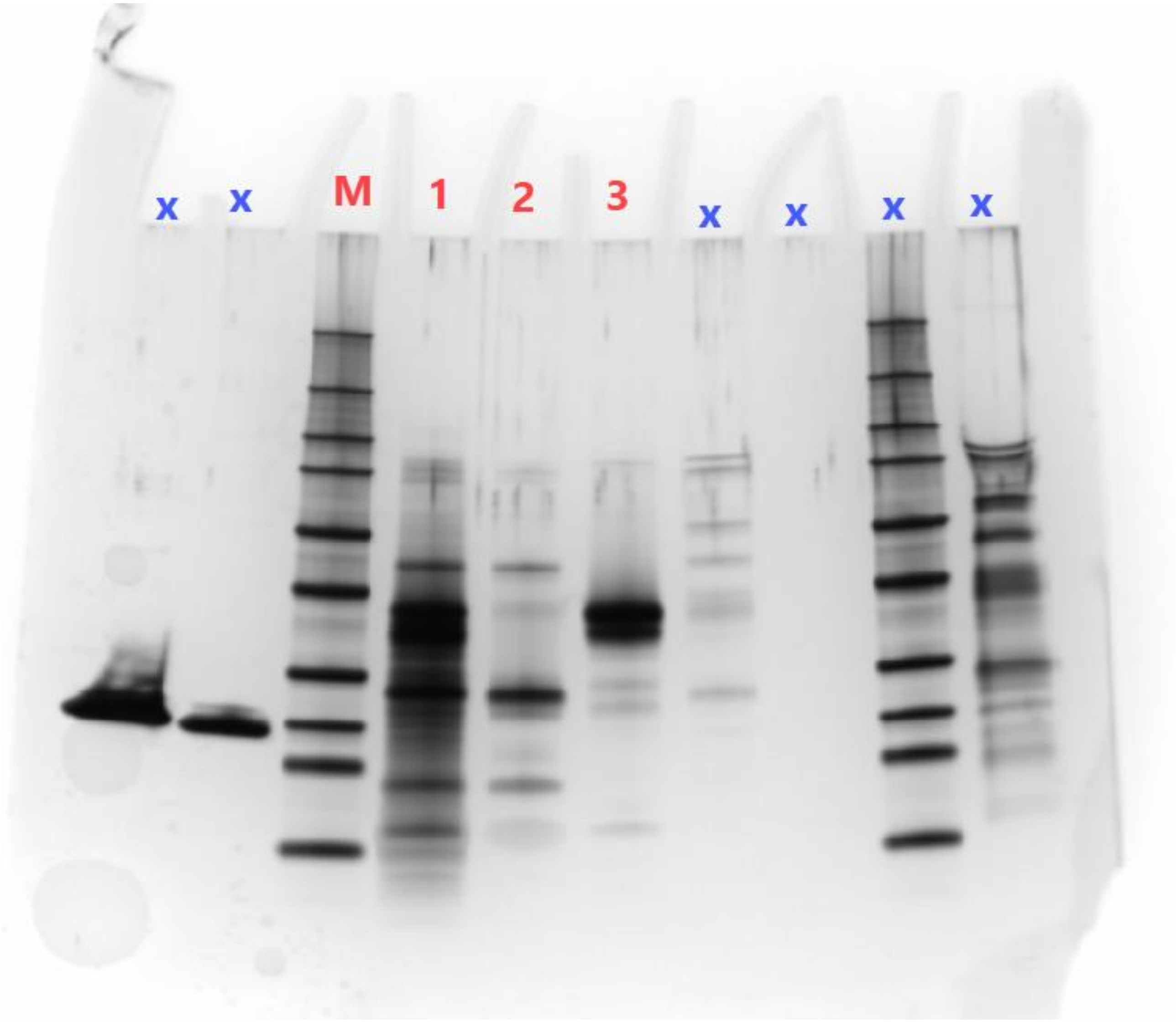

As indicated in Legend of Figure 1:

M are the molecular weight markers (BioRAD precision plus), whose exacts sizes are shown in Fig1

1, 2 and 3 are the samples shown in the manuscript, corresponding to:

- 1 – V.velutina extract, before application onto the first affinity chromatography
- 2 – Flow through of the elution
- 3 – Peak containing Vesp v 1, from same elution profile
- In all cases, **X** corresponds to unrelated samples with what is presented in the manuscript

SDSPAGE used in right panel of Fig1

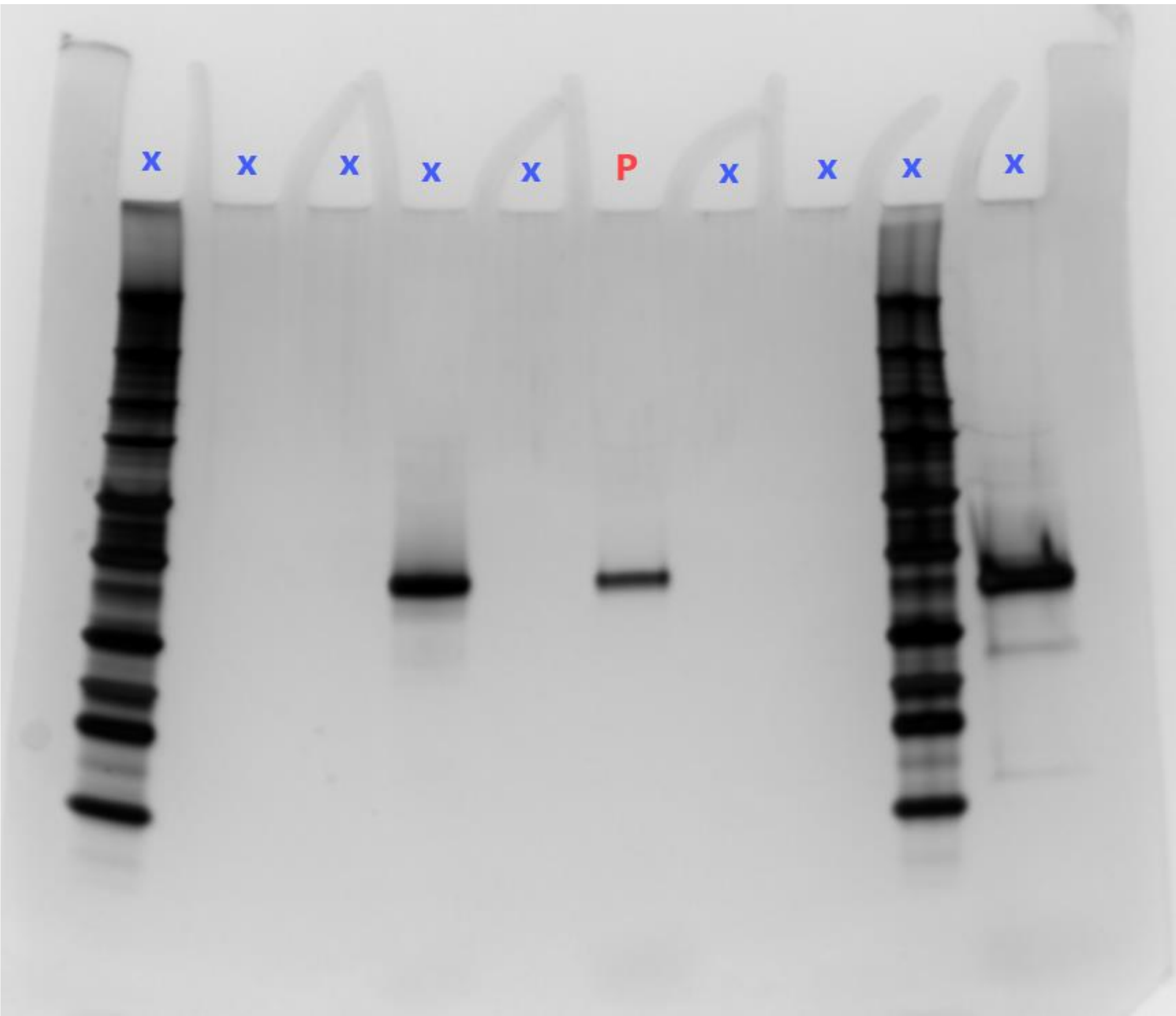

As indicated in Legend of Figure 1:

P corresponds to 0.25µg of purified Vesp v 1

(the figure is adjusted to left panel by aligning the molecular weight markers, and then the 2 panels were inserted into the Figure)

In all cases, X corresponds to unrelated samples with what is presented in the manuscript

SDSPAGE used in left panel of Fig2

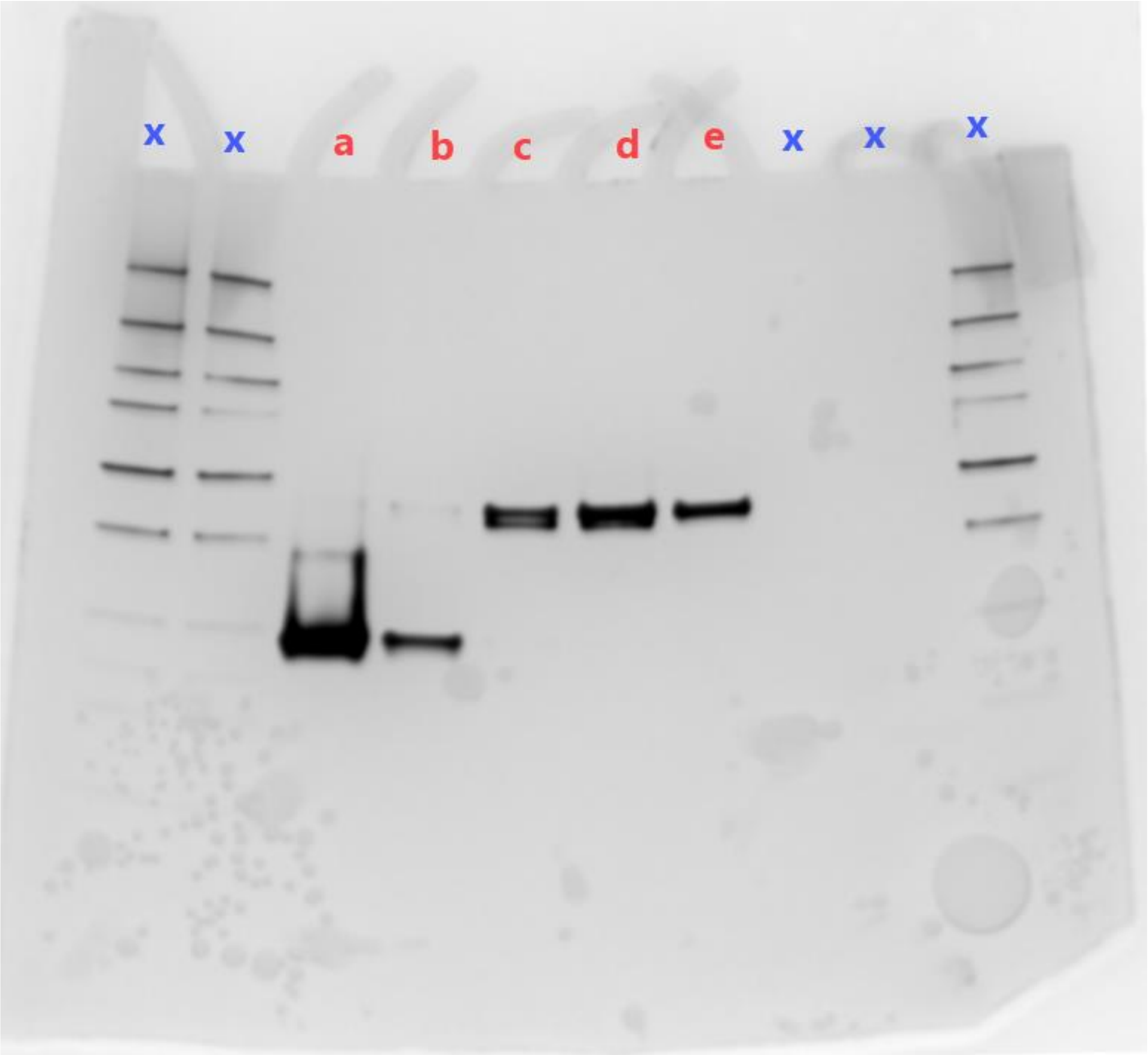

As indicated in Legend of Figure 2:

"**a, b, c, d, e**" correspond to successive fractions in the elution profile of the Heparin-Sepharose column, in order to follow the content of the peaks corresponding to Vesp v 5 and the Vesp v 2 isoforms.

- The exact position of the a,b,c,d,e fractions are shown in the chromatogram of Fig2

N.B. the silver-staining of this SDSPAGE did not reach its usual complete staining (that's why not all the bands in the MW markers are seen), but it is qualitatively very interesting:

- This was VERY USEFUL for us to see the main bands of the components being separated, and allowed us to perfectly observe the two isoforms of Vesp v 2 (see c,d,e fractions), that finally elute in a single broad peak.

X corresponds to unrelated samples with what is presented in the manuscript

SDSPAGE used in right panel of Fig2

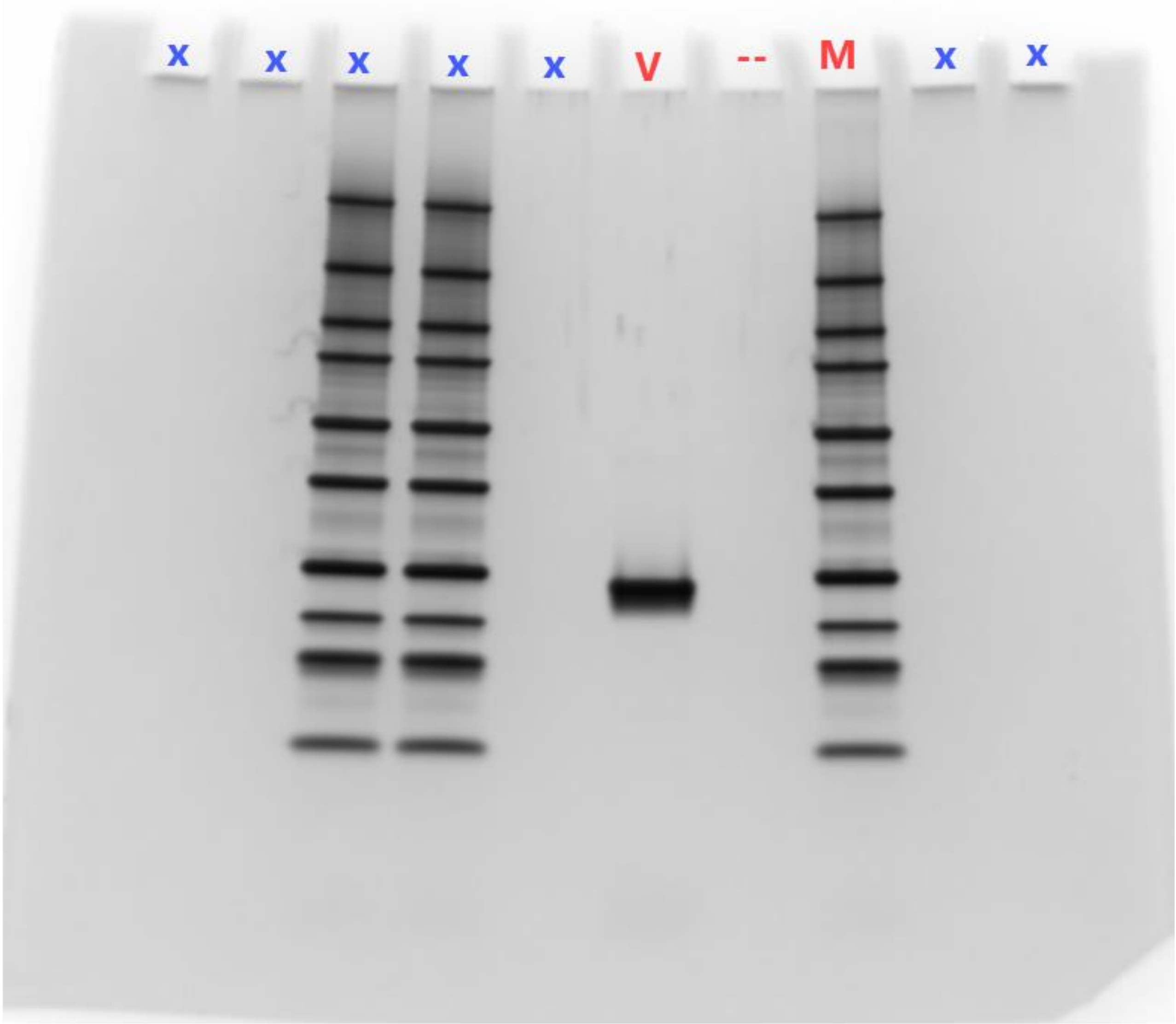

As indicated in Legend of Figure 2:

V corresponds to 0.25µg of purified Vesp v 5

"—" corresponds to well with "no sample", and

M corresponds to the MW markers (BioRad Precision Plus), and their size in kDa is then shown in the Figure2 insert.

X corresponds to unrelated samples with what is presented in the manuscript

(the figure is adjusted to left panel by aligning the molecular weight markers, and then the two panels were inserted into the Figure)
