# Supplementary material for: Purification and molecular characterization of phospholipase, antigen 5 and hyaluronidases from the venom of the Asian hornet (Vespa velutina)
Source: PLoS One. 2020 Jan 10;15(1):e0225672. doi: 10.1371/journal.pone.0225672 (PMC6953831; doi:10.1371/journal.pone.0225672)

PLOS ONE

Purification and molecular characterization of phospholipase, antigen 5 and hyaluronidases from the venom of the Asian hornet (Vespa velutina)

--Manuscript Draft--

**Manuscript Number:**	PONE-D-19-22139
**Article Type:**	Research Article
**Full Title:**	Purification and molecular characterization of phospholipase, antigen 5 and hyaluronidases from the venom of the Asian hornet (Vespa velutina)
**Short Title:**	Purification of Vespa velutina venom components
**Corresponding Author:**	RAFAEL IGNACIO MONSALVE, PHD<br>ALK-ABELLO<br>MADRID, SPAIN
**Keywords:**	VENOM PROTEIN PURIFICATION ASIAN HORNET VESPA VELUTINA SEQUENCE
**Abstract:**	The aim of this study was to purify ~~several~~ potential allergenic components of Vespa velutina venom, the yellow legged Asian Hornet, and perform a preliminary characterization of the purified proteins. Starting from the whole venom of V.velutina, several chromatographic steps allowed to purify the phospholipase (named Vesp v 1), as well as the antigen 5 (Vesp v 5, the only allergenic component described as such so far). The two hyaluronidase isoforms found (Vesp v 2A and Vesp v 2B) cannot be separated from each other, but they are partially purified and characterized. Purity of the isolated proteins in shown by SDSPAGE, as well as by the results of the N-terminal sequencing. nLC-MS/MS data complement most of the sequence for Vesp v 1 and Vesp v 5, confirming the data available in public transcriptomic databases, and this permits comparison with homologous sequences from other hymenoptera. The overall data confirm the high similarity of the V.velutina components with the analogous components of other Vespa and Vespula species.
**Order of Authors:**	RAFAEL IGNACIO MONSALVE, PHD
	RUTH GUTIERREZ
	ILKA HOOF
	MANUEL LOMBARDERO
**Additional Information:**	
**Question**	**Response**
**Financial Disclosure**<br><br>Enter a financial disclosure statement that describes the sources of funding for the work included in this submission. Review the submission guidelines for detailed requirements. View published research articles from *PLOS ONE* for specific examples.<br><br>This statement is required for submission and **will appear in the published article** if the submission is accepted. Please make sure it is accurate.	The author(s) received no specific funding for this work, apart from their regular salary.

Powered by Editorial Manager® and ProduXion Manager® from Aries Systems Corporation

**Unfunded studies**<br>Enter: *The author(s) received no specific funding for this work.*<br><br>**Funded studies**<br>Enter a statement with the following details:<br>• Initials of the authors who received each award<br>• Grant numbers awarded to each author<br>• The full name of each funder<br>• URL of each funder website<br>• Did the sponsors or funders play any role in the study design, data collection and analysis, decision to publish, or preparation of the manuscript?<br>• **NO** - Include this sentence at the end of your statement: *The funders had no role in study design, data collection and analysis, decision to publish, or preparation of the manuscript.*<br>• **YES** - Specify the role(s) played.<br><br>* typeset	
**Competing Interests**<br><br>Use the instructions below to enter a competing interest statement for this submission. On behalf of all authors, disclose any competing interests that could be perceived to bias this work—acknowledging all financial support and any other relevant financial or non-financial competing interests.<br><br>This statement **will appear in the published article** if the submission is accepted. Please make sure it is accurate. View published research articles from *PLOS ONE* for specific examples.	The authors have declared that no competing interests exist.

**NO authors have competing interests**<br><br>Enter: *The authors have declared that no competing interests exist.*<br><br>**Authors with competing interests**<br><br>Enter competing interest details beginning with this statement:<br><br>*I have read the journal's policy and the authors of this manuscript have the following competing interests: [insert competing interests here]*<br><br>* typeset	
**Ethics Statement**<br><br>Enter an ethics statement for this submission. This statement is required if the study involved:<br><br>• Human participants<br>• Human specimens or tissue<br>• Vertebrate animals or cephalopods<br>• Vertebrate embryos or tissues<br>• Field research<br><br>Write "N/A" if the submission does not require an ethics statement.<br><br>General guidance is provided below. Consult the submission guidelines for detailed instructions. **Make sure that all information entered here is included in the Methods section of the manuscript.**	N/A

**Format for specific study types**<br><br>**Human Subject Research (involving human participants and/or tissue)**<br>• Give the name of the institutional review board or ethics committee that approved the study<br>• Include the approval number and/or a statement indicating approval of this research<br>• Indicate the form of consent obtained (written/oral) or the reason that consent was not obtained (e.g. the data were analyzed anonymously)<br><br>**Animal Research (involving vertebrate animals, embryos or tissues)**<br>• Provide the name of the Institutional Animal Care and Use Committee (IACUC) or other relevant ethics board that reviewed the study protocol, and indicate whether they approved this research or granted a formal waiver of ethical approval<br>• Include an approval number if one was obtained<br>• If the study involved *non-human primates*, add *additional details* about animal welfare and steps taken to ameliorate suffering<br>• If anesthesia, euthanasia, or any kind of animal sacrifice is part of the study, include briefly which substances and/or methods were applied<br><br>**Field Research**<br><br>Include the following details if this study involves the collection of plant, animal, or other materials from a natural setting:<br>• Field permit number<br>• Name of the institution or relevant body that granted permission	
**Data Availability**<br><br>Authors are required to make all data underlying the findings described fully available, without restriction, and from the time of publication. PLOS allows rare exceptions to address legal and ethical concerns. See the PLOS Data Policy and FAQ for detailed information.	Yes - all data are fully available without restriction

A Data Availability Statement describing where the data can be found is required at submission. Your answers to this question constitute the Data Availability Statement and **will be published in the article**, if accepted.<br><br>**Important:** Stating 'data available on request from the author' is not sufficient. If your data are only available upon request, select 'No' for the first question and explain your exceptional situation in the text box.<br><br>Do the authors confirm that all data underlying the findings described in their manuscript are fully available without restriction?	
**Describe where the data may be found in full sentences. If you are copying our sample text, replace any instances of XXX with the appropriate details.**<br><br>• If the data are **held or will be held in a public repository**, include URLs, accession numbers or DOIs. If this information will only be available after acceptance, indicate this by ticking the box below. For example: *All XXX files are available from the XXX database (accession number(s) XXX, XXX.*).<br>• If the data are all contained **within the manuscript and/or Supporting Information files**, enter the following: *All relevant data are within the manuscript and its Supporting Information files.*<br>• If neither of these applies but you are able to provide **details of access elsewhere**, with or without limitations, please do so. For example:<br><br>*Data cannot be shared publicly because of [XXX]. Data are available from the XXX Institutional Data Access / Ethics Committee (contact via XXX) for researchers who meet the criteria for access to confidential data.*<br><br>*The data underlying the results presented in the study are available from (include the name of the third party*	PROTEIN SEQUENCES WILL BE AVAILABLE AT THE UNIPROT DATABASE (SEE TABLE 1 FOR ACCESSION NUMBERS)<br><br>Proteomic study data will be deposited and made available publicly when data are published with the corresponding ProteomeExchange identifiers. Data have been sent to the Mass spectrometry Interactive Virtual Enviroment (MassIVE).

and contact information or URL).<br>• This text is appropriate if the data are owned by a third party and authors do not have permission to share the data.<br><br>* typeset	
Additional data availability information:	Tick here if the URLs/accession numbers/DOIs will be available only after acceptance of the manuscript for publication so that we can ensure their inclusion before publication.

Powered by Editorial Manager® and ProduXion Manager® from Aries Systems Corporation

Manuscript

Click here to download Manuscript Purification Vespa velutina_MONSALVE.docx

Title page

Purification and molecular characterization of phospholipase, antigen 5 and hyaluronidases from the venom of the Asian hornet (*Vespa velutina*)

AUTHORS AND AFFILIATION

Rafael I. Monsalve[1*]; Ruth Gutiérrez[1], Ilka Hoof[2], Manuel Lombardero[1].

Affiliation:

(1) CMC Research and Development, ALK-Abelló S.A., Madrid, Spain.

(2) Global Research, ALK-Abelló A/S, Hørsholm, Denmark

(*) Rafael I. Monsalve is the corresponding author (Rafael.monsalve@alk.net)

Author Contributions

Formal Analysis: RIM, IH

Investigation: RIM, RG

Project Administration: ML

Writing of the paper: RIM

Writing – review and editing: RIM, ML

Data Availability Statement:

Proteomic study data will be deposited and made available publicly when data are published with the corresponding ProteomeExchange identifiers (data are being sent to the Mass spectrometry Interactive Virtual Enviroment (MassIVE).

The nomenclature of the allergen named Vesp v 5 is at this moment under discussion with the WHO/IUIS Committee. This allergenic protein was described in 2013-2015 by ML and other medical doctors, and the correct nomenclature is on its way of being confirmed (RM is taking direct charge of this)

Abstract

The aim of this study was to purify ~~several~~ potential allergenic components of *Vespa velutina* venom, the yellow legged Asian Hornet, and perform a preliminary characterization of the purified proteins. Starting from the whole venom of *V.velutina*, several chromatographic steps allowed to purify the phospholipase (named Vesp v 1), as well as the antigen 5 (Vesp v 5, the only allergenic component described as such so far). The two hyaluronidase isoforms found (Vesp v 2A and Vesp v 2B) cannot be separated from each other, but they are partially purified and characterized. Purity of the isolated proteins in shown by SDSPAGE, as well as by the results of the N-terminal sequencing. nLC-MS/MS data complement most of the sequence for Vesp v 1 and Vesp v 5, confirming the data available in public transcriptomic databases, and this permits comparison with homologous sequences from other hymenoptera. The overall data confirm the high similarity of the *V.velutina* components with the analogous components of other *Vespa* and *Vespula* species.

Introduction

The “yellow-legged”-Asian hornet (*Vespa velutina nigrithorax*) is an invasive species that was originally introduced in the Iberian peninsula through France, having been identified in Spain for the first time in 2010 [1]. Since then, it has extended its presence through the north of Spain, up to the north of Portugal, causing socio-economic concerns due to its important expansive capacity, and also considering that they

frequently attack honey-bee hives to feed their nests. Solutions for this invasion are being considered in different ways in several European countries [2-5]. From the allergologic point of view, it is also a concern, as demonstrated in the North of Spain [6], when the first cases of anaphylactic shocks were described, and when “antigen 5”, from the venom of these Asian hornets, was identified as the most important allergen in a group of eight sensitized patients. Since then, they have increased its presence, mostly in North-Western parts of the Spanish coast.

The data we have obtained from these purified components, confirm the whole sequence data available in transcriptomic databases since 2015 [7], and this allows a thorough comparison with homologous components from other hymenoptera. Moreover, the purified components will be used to demonstrate their relevance as allergens, by Component Resolved Diagnosis (CRD) in studies performed in platforms that can measure specific IgE in the sera of allergic patients ([8;9]), which are useful to differentiate which can be the sensitizing species for each patient, as we also published in the past ([10]), differentiating on *Vespula* vs. *Polistes* sensititization.

The nomenclature used for naming the purified components, will consider what is suggested for allergenic proteins by the WHO/IUIS (World Health Organization / International Union of Immunological Societies) Committee ([11;12]), considering that only the antigen 5 (named Vesp v 5) has been described as such; the phospholipase (Vesp v 1) and hyaluronidases (Vesp v 2) must still be considered as potential allergenic components.

In summary, in this work we isolate the main potential allergenic components of the *V.velutina* venom, and show the potential interest on the availability of these purified

components: their characterization allow a closer understanding of similarities among different hymenoptera venom components, and will surely be useful in a better diagnosis and treatment of allergic patients to stings of this Asian hornet.

Materials and Methods

The relevant natural venom components (A1 phospholipase, antigen 5 and hyaluronidases) were purified lyophilized *Vespa velutina* venom sac extract of individual hornets collected in Europe (ALK Source Materials Inc., Spring Mills, batch 01071301AH). The purification was performed as previously described ~~in~~ [13;14], and in a similar manner as performed with other natural allergens from *Vespula vulgaris* and *Polistes dominula* [10]. The purified proteins were analysed by SDS–PAGE (Novex-Tricine, Invitrogen, UK) and silver- or Coomassie blue-stained, depending on the needs of the analysis (SDSPAGE ~~is~~ silver stained, according to [15]). The enzymatic activity of phospholipase and hyaluronidases was verified when needed by the methods of Habermann [16] and Richman and Baer [17], respectively, using *Apis mellifera* venom preparation as reference.

The purity and identity of the purified proteins was also confirmed, according to [18], by N-terminal sequencing analyses (direct analysis from the protein in solution), performed at CIB Protein Chemistry Service (CSIC, Madrid, Spain).

In the case of Vesp v 1 and Vesp v 5 additional nLC-MS/MS (nano Liquid Chromatography tandem mass spectrometry) analyses were performed from the bands extracted from an SDSPAGE stained with Coomassie blue: this protein

identification by nLC-MS/MS was carried out in the Proteomics and Genomics Facility (CIB-CSIC, Madrid - Spain), a member of ProteoRed-ISCIII network, according to the method described in [19]. For the MS analysis, Peptides were trapped onto a Acclaim PepMap 100 (Thermo Scientific) precolumn, and then eluted onto a columna Acclaim PepMap 100 C18 column, inner diameter 75 µm, 25 cm long, 3 µm particle size (Thermo Scientific) and separated using a 130 min gradient (100 min from 0% -35% Buffer B; 20 min from 35% -45% Buffer B ; 5 min from 45% -95% Buffer B; 4min 95% Buffer B and 1 min 0% Buffer B; (Buffer A: 0.1% formic acid, 2% acetonitrile and Buffer B: 0.1% formic acid in acetonitrile) at a flow-rate of 250 nL/min on a nanoEasy HPLC (Proxeon) coupled to a nanoelectrospray (Thermo Scientific). Mass spectra were acquired on a LTQ-Orbitrap Velos mass spectrometer (Thermo-Scientific) in the positive ion mode. Full-scan MS spectra (m/z 300-18000) were acquired in the Orbitrap at a resolution of 60,000 and the 15 most intense ions were selected for collision induced dissociation (CID) fragmentation in the linear ion trap with a normalized collision energy of 35%. Singly charged ions and unassigned charge states were rejected. Dynamic exclusion was enabled with exclusion duration of 45 s.

MS data were analysed according to [19], Mass spectra raw files were searched against an in –house specific database with known venom allergen sequences and sequences extracted from transcriptomic databases, using the Sequest search engine through Proteome Discoverer (version 1.4.1.14) (Thermo Scientific). Search parameters included a maximum of two missed cleavages allowed, carbamidomethyl of cysteines as a fixed modification and oxidation of methionine as variable modifications. Precursor and fragment mass tolerance were set to 10 ppm and 0.5 Da, respectively.

Identified peptides were validated using Percolator algorithm with a q-value threshold of 0.01.

The publicly available transcriptomic data for the Asian hornet *Vespa velutina nigrithorax* [7] was downloaded from the GEO database (SRA experiment SRX595647). The sequence data was originally obtained from RNA extracted from the venom glands of Asian hornet species collected in China ([7]). Using the FASTX-Toolkit (http://hannonlab.cshl.edu/fastx_toolkit/), the sequencing reads were trimmed and filtered with respect to quality, trimming bases from the ends with quality score below 20 and keeping reads that were at least 50 bases long after trimming, resulting in 203,886,099 reads (99.9% of the original data set). *De novo* transcriptome assembly was performed using the Trinity software (v2.1.1) [20]. From the assembled transcript sequences, the "coding sequence" or CDS (sequences corresponding to the natural proteins in the Open Reading Frames) can be extracted for further analyses.

Nomenclature used for the potential allergenic molecules of *Vespa velutina* venom, follow the rules recommended by the WHO/IUIS Committee ([11;12]). Protein parameters from the protein sequences (e.g. molecular weight calculation, extinction coefficients, prediction of N-glycosylation, etc.) and alignments were obtained from the software package at the Expasy Bioinformatic portal at the SIB (Swiss Institute of Bioinformatics) [21]. Furthermore, sequence homology searches were performed with the program BLAST (Basic Local Alignment Tool) [22]) provided by the NCBI (National Centre of Biotechnology Information, U.S. National Library of Medicine, Bethesda, USA).

Results and Discussion

Vesp v 1, the A1-phospholipase from the venom sac extract was purified as in [13], by directly using as specific affinity chromatography (Fig 1) followed by an ion exchange chromatography (Mono-S column, GE Healthcare). The flowthrough (FT) from the previous affinity chromatography was applied onto a Heparin-Sepharose column (Fig 2), which permits the purification of Vesp v 5 and of two isoforms of Hyaluronidase (namely Vesp v 2A and 2B).

Fig 1 – Elution profile of PC-Sepharose and SDSPAGE analysis. This affinity chromatography shows the initial separation of the components of the *Vespa velutina* extract. The continuous line corresponds to absorbance at 280nm (left axis, where "mAU" stands for milli-absorbance units). The parallel dotted line corresponds to the absorbance at 215nm. The right axis corresponds to pH units (curve shown with discontinuous dashes), and it can be observed that the phospholipase elution occurs when the pH decreases (the Vesp v 1 peak, as indicated by an arrow). The rest of the venom components elute in the "Flowthrough" (FT, shown as a horizontal double-arrow. The SDSPAGE in the inset shows the proteins separated in this step: Lane 1 corresponds to the initial extract, lane 2 corresponds to the FT, and lane 3 to the Vesp v 1 peak (Peak). Lane P shows the purified Vesp v 1 (0.25μg), after the final ion-exchange chromatography on a mono-S column. Lane M corresponds to the molecular weight markers (BioRad Precision Blue), and their size in kDa are shown on the left margin of the SDSPAGE inset. SDSPAGE shown are silver stained ([15]).

Fig 2 – Elution profile of Heparin-Sepharose column and SDSPAGE analysis.

This chromatographic profiles shows the effect of the NaCl gradient (the dotted line showing the conductivity, in mS/cm, as shown in the right scale), and the separation of the components coming from the FT of the chromatography shown in Fig 1: Vesp v 5 elutes as the major peak, clearly separated from the Vesp v 2 hyaluronidases. In the inserted SDSPAGEs, the left panel shows consecutive fractions of the chromatography (fractions a, b, c, d, e).

Besides, the right panel shows the purified Vesp v 5 (lane V, 0.25µg), a blank lane (-) and molecular weight markers (lane M); the molecular weight in kDA for the BioRad Precision Blue are shown on the right margin of the silver-stained SDSPAGE.

The proteins purified have been analyzed by direct N-terminal protein sequencing (Edman degradation), showing the results presented in Table 1. In the case of Vesp v 2, it is clear that there are two proteins, and their sequences correspond to the first 10 residues of each of the isoforms found in the *Vespa velutina* transcriptome databases (Fig 3); moreover, one of the isoforms is clearly the most abundant (isoform B). The data obtained in the N-terminal sequencing also demonstrates that the Asn in 3rd position of isoform A must be glycosylated (wasps and hornet hyaluronidases are known to be glycosylated; e.g. Ves v 2 in known to contain at least 3 glycosylation sites [23]). This is also confirmed by the apparent molecular weight exhibited by the two isoforms (40.0-41.7kDa, as calculated from the SDS-PAGE, Fig 2), which are higher than the 39.1 and 40.0kDa values that correspond to the polypeptide part of the complete "coding sequence" or CDS (Fig 3) of isoforms A and B, respectively (Protein Parameters determined in [21]). Fig 3 also shows the positions of the predicted glycosylation sites for each isoform of Vesp v 2 (four potential sites in each of them). An additional test performed in some fractions of the chromatogram shown in Fig 3 was the specific

activity for hyaluronidase by the method of Richman ([17]): the semi-quantitative result indicated that fractions “c”, “d” and “e” had clear hyaluronidase activity.

Table 1 – N-terminal sequences determined for the purified proteins of the venom of *V.velutina*.

The sequential result of the amino acids resulting from the Edman degradation are shown (in the case of Vesp v 1 and Vesp v 5 only 5 sequencing cycles were performed). In the column at right, the corresponding accession numbers assigned to each protein in the UniProt Knowledgebase are shown (they will contain all the sequence data published in this paper). The purified proteins are named considering their potential allergenic nomenclature, even if the only major allergen confirmed to date is Vesp v 5, as approved by the WHO/IUIS Committee ([11;12]).

**Protein**	**N-terminal sequencing results**									**UNIPROT #**
Vesp v 2A	Asn	Leu	(*) Asn	Arg	Thr	Asn	Trp	Pro	Lys	Lys…	C0HLL4
Vesp v 2B	Ser	Glu	Arg	Pro	Lys	Arg	Val	Phe	Asn	Ile…	C0HLL5
Vesp v 1	Gly	Leu	Leu	Pro	Lys…						C0HLL3
Vesp v 5	Asn	Asn	Tyr	Cys	Lys…						P0DMB9

(*) Asn derivative corresponding to N-glycosylation

Fig 3 – **Complete coding sequence of hyaluronidases isoforms (Vesp v 2A and Vesp v 2B).** Panels A and B show the CDS of extracted from the transcriptomic data of *Vespa velutina* ([7;20]) for Vesp v 2A (341 amino acids, 40012.2 Da) and Vesp v 2B (331 amino acids, 39109.7 Da), respectively. Numbers above the sequences facilitate the localization of the amino acids

(the numbers are positioned every 10th residue). In both panels, horizontal lines with arrows located below the sequences indicate the “potential N-glycosylation sites” for each sequence (defined as Asn-Xaa-Ser/Thr sequons, according to the prediction performed at the NetNGlyc 1.0 Server [21]). The only confirmed glycosylated residue (marked with an asterisk below the sequence) is Asn-3 in the Vesp v 2A isoform, as shown in the data presented in Table 1. Besides, in the referred table, the accession numbers assigned to these sequences in the UNIPROT database are shown.

The N-terminal sequence data for Vesp v 1 and Vesp v 5 show a unique sequence, confirming its purity. Besides their direct analysis by nLC-MS/MS also confirm that most of the peptides found in this analyses correspond exactly to the sequences extracted from the *Vespa velutina* transcriptomic data ([7]), as shown in Fig 4. As it can be observed, 72% and 87% coverage, for Vesp v 1 and Vesp v 5 respectively, have been experimentally determined by this combination of techniques. An interesting feature for Vesp v 1, is that it is a glycosylated phospholipase; this can be deducted from the fact that nLC-MS/MS studies have not provided any data on the peptide in which Asn-61 is potentially predicted as N-glycosylated (Fig 4A); on the contrary, Asn-40 is not glycosylated, since the peptide containing this amino acid is clearly identified by nLC-MS/MS (Fig 4A). This is therefore another peculiar A1-phospholipase, since they are not usually glycosylated (so far, this had been only described for two phospholipases: Dol m I from *Dolichovespula maculata* ([24]) and for Sol i 1, from the imported fire ants ([25]). Moreover, the glycosylation of Vesp v 1 is also confirmed by the higher apparent molecular weight this protein exhibits in an SDSPAGE (36.1kDa, Fig 1), in

comparison with the 34.0kDa that correspond to the polypeptide part of its complete CDS (Figure 4).

Fig 4 – Complete coding sequence of Vesp v 1 and Vesp v 5, and characterization of its sequences. Panel A shows the Vesp v 1 CDS extracted from the trancriptomic data of *Vespa velutina* (304 amino acids, 33957.2 Da), highlighting in yellow the peptides identified by nLC-MS/MS analysis, and in green the N-terminal sequence determined (see Table 1). Moreover the two potential N-glycosylation sites for Asn ([21]) are also indicated by horizontal arrows, but only the second predicted site seems to be glycosylated (Asn-61, as explained in the text). Panel B shows the CDS for Vesp v 5 (202 amino acids, 22717.7 Da), similarly coloured as in panel B for Vesp v 1; no N-glycosylation is predicted for this antigen 5. These complete sequence data are available at UNIPROT database, with the accession numbers indicated in Table 1.

These complete CDS, corresponding to relevant proteins present in the venom of *Vespa velutina*, have been assigned the corresponding UNIPROT accession numbers, as shown in Table 1, and are now available for further comparison with other homologous venom components described to date. Moreover, complete sequence data are also sent to theWHO/ IUIS-Allergen-Nomenclature committee (www.allergen.org), so that the correct nomenclature is used for the components that have been shown as relevant allergens, as it is the case of Vesp v 5, already defined as a major allergen [6], but that since now it will contain the complete CDS for this allergen. Vesp v 5 exhibits an apparent molecular weight of 23.5kDa in SDSPAGE (Fig

2), close to the 22.7 kDa calculated size from the complete CDS shown in Figure 4 (as expected, antigen 5 is not a glycosylated component).

The major allergen Vesp v 5, and the potential major allergen Vesp v 1, have been purified to homogeneity, and most of their CDS determined (with an 87% and 74% of total coverage, respectively; Figure 4), matching perfectly with the extracted data from the *Vespa velutina* transcriptome [7]. This also confirms that these sequence data, obtained from the RNA extracted from the venom glands of the hornet species present in China [7], match exactly with the experimental data determined from the isolated natural components from the venom from species present in Europe, where the source material was collected. In the case of the hyaluronidases isoforms (named Vesp v 2A and Vesp v 2B), the complete CDS have not been extensively characterized experimentally, but the exact match of the first 10 amino acid residues of each isoform (Figure 3 / Table 1) permits to reasonably think that the whole CDS will be those extracted from the complete transcriptomic database. Nevertheless, the partial characterization of these hyaluronidases isoforms, is completed, apart from the purity shown in the chromatographic peak containing the aforementioned isoforms (Fig 2), but also considering that the enzymatic activity for hyaluronidase was specifically measured in fractions “c”, “d” and “e” shown in Fig 2, and as it was measured when the *Vespula vulgaris* hyaluronidase (Ves v 2) was purified by Monsalve et al. [10]. Moreover, our data on the Vesp v 2B isoform, as the most abundant hyaluronidase, is coincident with the data presented by Kolarich et al. [26] for the isoforms of *Vespula vulgaris*.

These complete CDS allow an overall sequence comparison with the previously known homologous allergen components of diverse hymenoptera species, being of interest the similarity with other *Vespa*, *Vespula* or *Polistes* species. A remarkable similarity has been found between the components of *Vespa* and *Vespula*, much higher than with regard to *Polistes*. It is particularly striking, as shown in Fig 5, the fact that one of the hyaluronidase isoforms of *Vespa* and *Vespula* exhibit 93% sequence identity (and only 73% in the case of the isoform present in *Polistes*), and that the other isoform exists in *Vespula* and *Vespa* and it is not present at all in *Polistes* (these data can be confirmed by having compared with the sequences available to the date in the existing public sequence databases of both nucleotide and protein sequences, searched with the program BLAST (Basic Local Alignment Tool) [22]).

Fig 5 – Alignment of several hymenoptera hyaluronidases with respect to Vesp v 2 isoforms. Panel A- Alignment of Vesp v 2 isoforms with the sequences from relevant species available in sequence-databases. The multiple sequence alignment is performed by the program ClustalW ([27]). In the overall alignment, the upper sequence corresponds to Vesp v 2A, and below that, a dot represents identity with the upper sequence, and a dot surrounded in a black square indicates absolute identity among all the aligned proteins (when there is no identity, the corresponding amino acid is shown); finally, dashes correspond to the gaps opened by the ClustalW alignment program. On the right margin of the sequences, the corresponding numbering of each sequence is shown. The sequences compared with the two new *Vespa velutina* isoforms, shown as Vesp_v_2A and Vesp_v_2B, and shown in the

alignment are: two isoforms from *Vespula germanica*, Ves g 2A (Uniprot Q05FZ1) and Ves_g_2B (Uniprot Q05FZ2), and the hyaluronidases found for *Vespa magnifica* (Vesp_mg_2, Uniprot P86875) and *Polistes dominula* (Pol_d_2; NCBI Reference XP_015179722). In all cases, mature proteins are compared (i.e. starting from the N-terminal of the corresponding protein).

Panel B summarizes the identity percentages among these homologous hyaluronidases aligned, differentiating the very high similarities of Vesp v 2B with *Vespa* and *Vespula*'s hyaluronidase (above), and the somehow lower similarities versus (vs) the Vesp v 2A isoform.

The similarities between the different genera, comparing their phospholipases and antigen 5s are shown in Fig 6 and Table 2, and show the highest similarity of the Asian hornet components with those of the *Vespa* and *Vespula* species, in comparison with those of the *Polistes* species.

Fig 6 – Multiple sequence alignment of Vesp v 1 and Vesp v 5 with other homologous hymenoptera components.

Panel (A) and (B) show the alignment of the complete CDS for phospholipases and antigen 5s respectively. The complete mature Vesp v 1 and Vesp v 5 sequences are ~~are~~ shown, and are compared with the corresponding mature sequences of a homologous representative from *Vespa crabro*, *Vespula* vulgaris and *Polistes dominula*. The colouring scheme for the alignments is the same than in Fig 5A. On the right margin of the sequences, the corresponding numbering of each sequence is shown. In panel (A) the accession codes of the proteins compared with Vesp v 1 are: Vesp_c_1 (Uniprot P0CH87), Ves_v_1 (Uniprot P49369) and Pol_d_1 (Uniprot

Q6Q252). The accession codes of the proteins compared with Vesp v 5 (panel B) are: Vesp_c_5 (Uniprot P35782), Ves_v_5 (EMBL CAB42887.1) and Pol_d_5 (Uniprot P81656).

Table 2 - Sequence Identity and Similarity of Vesp v 1 and Vesp v 5 in comparison with the homologous components of other hymenoptera. Data are obtained after the sequence alignments shown in Fig 6, the values shown correspond to the ~~per cent~~ identity, and the ~~per cent~~ similarity is shown in brackets (similarity refers to residues having total identity between the sequences, plus those amino acids that are considered to be conservative substitutions as explained in [27]).

Identiy % (similarity %) with respect to *Vespa velutina* components		
	**Phospholipases**	**Antigen 5s**
Vespa crabro	69 (78)	88 (93)
Vespula vulgaris	61 (76)	66 (77)
Polistes dominula	53 (71)	58 (75)

The availability of the purified components from *V.velutina* venom will allow to confirm their individual relevance as allergens in patients sensitized to *V.velutina* venom. Vesp v 5 had already been described as the major allergen of *V.velutina* venom [6], but the phospholipase Vesp v 1 cannot be discarded also as a major allergen in hymenoptera-sensitized patients, as previously shown in several studies in which these two allergenic components have been shown to differently affect different allergic patients [10] and also considering the described intrinsic importance as allergens of

phospholipases [28]. These pure components (Vesp v 1 and Vesp v 5) have been specifically prepared as “detection reagents” for measuring sIgE, following the procedure described by Erwin et al. [29] and will be tested in patients that have shown anaphylactic shocks after being stung by *V.velutina*.

The clinical data that will be obtained in future experiments with the use of these isolated components, as well as the overall data of the clinical history of patients being already treated with different commercially available preparations, will confirm if the clear similarity existing among *Vespa* and *Vespula* components justifies that the treatment of these patients with *Vespula*-containing preparations is really being effective in protecting these patients, as already shown for *Vespa* spp.-sensitized patients [30;31], that had been protected by immunotherapy with *Vespula* commercial preparations, in contrast with the opinion of other authors who consider that a specific treatment would be more adequate [32], even if they mainly refer to safety issues.

More biochemical and immunological studies, including component-resolved-diagnosis with the proteins purified in this work and other components available, are therefore needed to confirm which will be the best treatment for these sensitized patients, also considering that the patients being treated nowadays are very probably patients that have become allergic after stings of the wasps and hornets present in these areas until now (*Vespula*, *Polistes* and/or *Vespa crabro*), but there might be a completely new population of patients who will develop allergenicity by *Vespa velutina* stings as the culprit insect.

Conclusions

~~Several~~ important components from the venom of *Vespa velutina* (Asian hornet) have been purified and partially characterized. Apart from Vesp v 5, the antigen 5 already described as a major allergen, also the A1-phospholipase and two hyaluronidases isoforms have been purified (they are potentially allergenic components, named Vesp v 1, and Vesp v 2A and Vesp v 2B, respectively).

The complete coding sequences (CDS) of these components are now available, after this characterization and having been extracted their CDS from the transcriptome data available for the venom glands of *V.velutina*.

The purified components will be used to further characterize the sensitization of patients that show anaphylactic shocks after being stung by these Asian hornets, which have invaded several European countries, and treat these patients in the best way possible.

Acknowledgments

We acknowledge the accurate work performed by the CIB Protein Chemistry Service (CSIC Madrid – Spain), specifically that of Dr.Varela and his team, for the analyses carried out to characterize the purified proteins shown in this work.

References

(1) Castro L, Pagola-Carte S. Vespa velutina Lepeletier, 1836 (Hymenoptera: Vespidae), collected in the Iberian Peninsula. Heteropterus Rev Entomol 2010;10(2):193-6.

(2) Leza M, Herrera C, Marques A, Roca P, Sastre-Serra J, Pons DG. The impact of the invasive species Vespa velutina on honeybees: A new approach based on oxidative stress. Sci Total Environ 2019 Jul 1;689:709-15.

(3) Milanesio D, Saccani M, Maggiora R, Laurino D, Porporato M. Recent upgrades of the harmonic radar for the tracking of the Asian yellow-legged hornet. Ecol Evol 2017 Jul;7(13):4599-606.

(4) Villemant C, Zuccon D, Rome Q, Muller F, Poinar GO, Jr., Justine JL. Can parasites halt the invader? Mermithid nematodes parasitizing the yellow-legged Asian hornet in France. PeerJ 2015;3:e947.

(5) Arca M, Papachristoforou A, Mougel F, Rortais A, Monceau K, Bonnard O, et al. Defensive behaviour of Apis mellifera against Vespa velutina in France: testing whether European honeybees can develop an effective collective defence against a new predator. Behav Processes 2014 Jul;106:122-9.

(6) Chugo S, Lizaso MT, Alvarez MJ, Arroabaren E, Lizarza S, Tabar AI. Vespa velutina nigritorax: A New Causative Agent in Anaphylaxis. J Investig Allergol Clin Immunol 2015;25(3):231-2.

(7) Liu Z, Chen S, Zhou Y, Xie C, Zhu B, Zhu H, et al. Deciphering the venomic transcriptome of killer-wasp Vespa velutina. Sci Rep 2015;5:9454.

(8) Schiener M, Graessel A, Ollert M, Schmidt-Weber CB, Blank S. Allergen-specific immunotherapy of Hymenoptera venom allergy - also a matter of diagnosis. Hum Vaccin Immunother 2017 Oct 3;13(10):2467-81.

(9) Antolin-Amerigo D, Ruiz-Leon B, Boni E, Alfaya-Arias T, Alvarez-Mon M, Barbarroja-Escudero J, et al. Component-resolved diagnosis in hymenoptera allergy. Allergol Immunopathol 2017 Jul 21;46(3):253-62.

(10) Monsalve RI, Vega A, Marques L, Miranda A, Fernandez J, Soriano V, et al. Component-resolved diagnosis of vespid venom-allergic individuals: phospholipases and antigen 5s are necessary to identify Vespula or Polistes sensitization. Allergy 2012 Apr;67(4):528-36.

(11) King TP, Hoffman D, Lowenstein H, Marsh DG, Platts-Mills TA, Thomas W. Allergen nomenclature. WHO/IUIS Allergen Nomenclature Subcommittee. Int Arch Allergy Immunol 1994;105(3):224-33.

(12) Pomes A, Davies JM, Gadermaier G, Hilger C, Holzhauser T, Lidholm J, et al. WHO/IUIS Allergen Nomenclature: Providing a common language. Mol Immunol 2018 Aug;100:3-13.

(13) King TP, Kochoumian L, Joslyn A. Wasp venom proteins: phospholipase A1 and B. Arch Biochem Biophys 1984;230(1):1-12.

(14) King TP, Lu G, González M, Qian NF, Soldatova L. Yellow jacket venom allergens, hyaluronidase and phospholipase: sequence similarity and antigenic cross-reactivity with their hornet and wasp homologs and possible implications for clinical allergy. Journal of Allergy and Clinical Immunology 1996;98:588-600.

(15) Morrissey JH. Silver stain for proteins in polyacrylamide gels: a modified procedure with enhanced uniform sensitivity. Anal Biochem 1981;117(2):307-10.

(16) Habermann E, Hardt KL. A sensitive and specific plate test for the quantitation of phospholipases. Anal Biochem 1972;50(1):163-73.

(17) Richman PG, Baer H. A convenient plate assay for the quantitation of hyaluronidase in Hymenoptera venoms. Anal Biochem 1980;109(2):376-81.

(18) Speicher KD, Gorman N, Speicher DW. N-terminal sequence analysis of proteins and peptides. Curr Protoc Protein Sci 2009 Aug;Chapter 11:Unit11.

(19) Kall L, Canterbury JD, Weston J, Noble WS, MacCoss MJ. Semi-supervised learning for peptide identification from shotgun proteomics datasets. Nat Methods 2007 Nov;4(11):923-5.

(20) Haas BJ, Papanicolaou A, Yassour M, Grabherr M, Blood PD, Bowden J, et al. De novo transcript sequence reconstruction from RNA-seq using the Trinity platform for reference generation and analysis. Nat Protoc 2013 Aug;8(8):1494-512.

(21) Artimo P, Jonnalagedda M, Arnold K, Baratin D, Csardi G, de CE, et al. ExPASy: SIB bioinformatics resource portal. Nucleic Acids Res 2012 Jul;40(Web Server issue):W597-W603.

(22) Altschul SF, Gish W, Miller W, Myers EW, Lipman DJ. Basic local alignment search tool. J Mol Biol 1990 Oct 5;215(3):403-10.

(23) Skov LK, Seppala U, Coen JJ, Crickmore N, King TP, Monsalve R, et al. Structure of recombinant Ves v 2 at 2.0 Angstrom resolution: structural analysis of an allergenic hyaluronidase from wasp venom. Acta Crystallogr D Biol Crystallogr 2006;62(Pt 6):595-604.

(24) Soldatova L, Kochoumian L, King TP. Sequence similarity of a hornet (D. maculata) venom allergen phospholipase A1 with mammalian lipases. FEBS Lett 1993;320(2):145-9.

(25) Hoffman DR, Sakell RH, Schmidt M. Sol i 1, the phospholipase allergen of imported fire ant venom. J Allergy Clin Immunol 2005 Mar;115(3):611-6.

(26) Kolarich D, Loos A, Leonard R, Mach L, Marzban G, Hemmer W, et al. A proteomic study of the major allergens from yellow jacket venoms. Proteomics 2007;7(10):1615-23.

(27) Thompson JD, Higgins DG, Gibson TJ. CLUSTAL W: improving the sensitivity of progressive multiple sequence alignment through sequence weighting, position-specific gap penalties and weight matrix choice. Nucleic Acids Res 1994 Nov 11;22(22):4673-80.

(28) Perez-Riverol A, Lasa AM, Dos Santos-Pinto JRA, Palma MS. Insect venom phospholipases A1 and A2: Roles in the envenoming process and allergy. Insect Biochem Mol Biol 2019 Feb;105:10-24.

(29) Erwin EA, Custis NJ, Satinover SM, Perzanowski MS, Woodfolk JA, Crane J, et al. Quantitative measurement of IgE antibodies to purified allergens using streptavidin linked to a high-capacity solid phase. J Allergy Clin Immunol 2005 May;115(5):1029-35.

(30) Goldberg A, Shefler I, Panasoff J, Paitan Y, Confino-Cohen R. Immunotherapy with commercial venoms is efficacious for anaphylactic reactions to Vespa orientalis stings. Int Arch Allergy Immunol 2013;161(2):174-80.

(31) Kosnik M, Korosec P, Silar M, Music E, Erzen R. Wasp venom is appropriate for immunotherapy of patients with allergic reaction to the European hornet sting. Croat Med J 2002;43(1):25-7.

(32) Macchia D, Cortellini G, Mauro M, Meucci E, Quercia O, Manfredi M, et al. Vespa crabro immunotherapy versus Vespula-venom immunotherapy in Vespa crabro allergy: a comparison study in field re-stings. World Allergy Organ J 2018;11(1):3.

Figure1

Click here to download Figure Fig1.tif

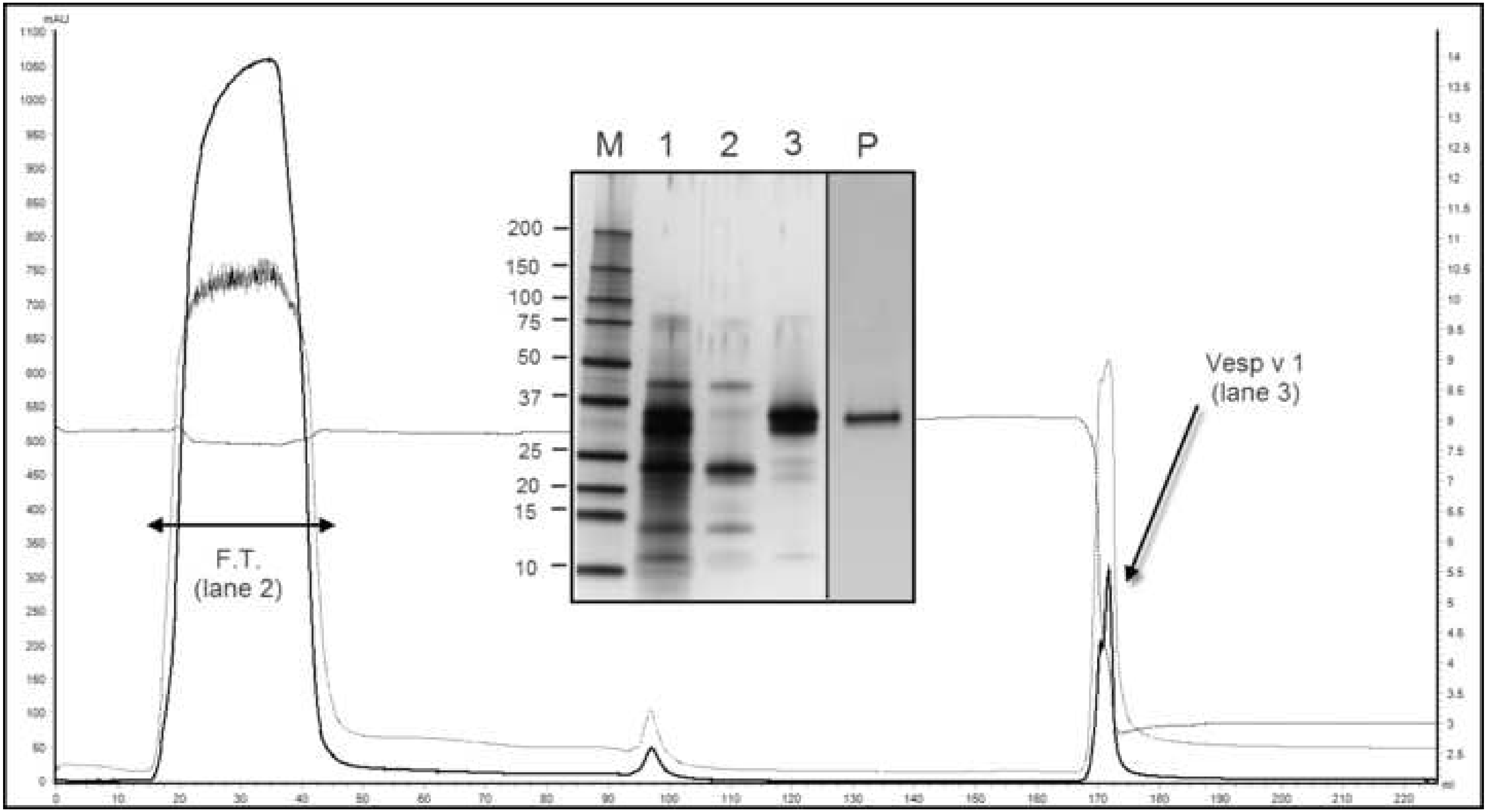

Figure2

Click here to download Figure Fig2.tif

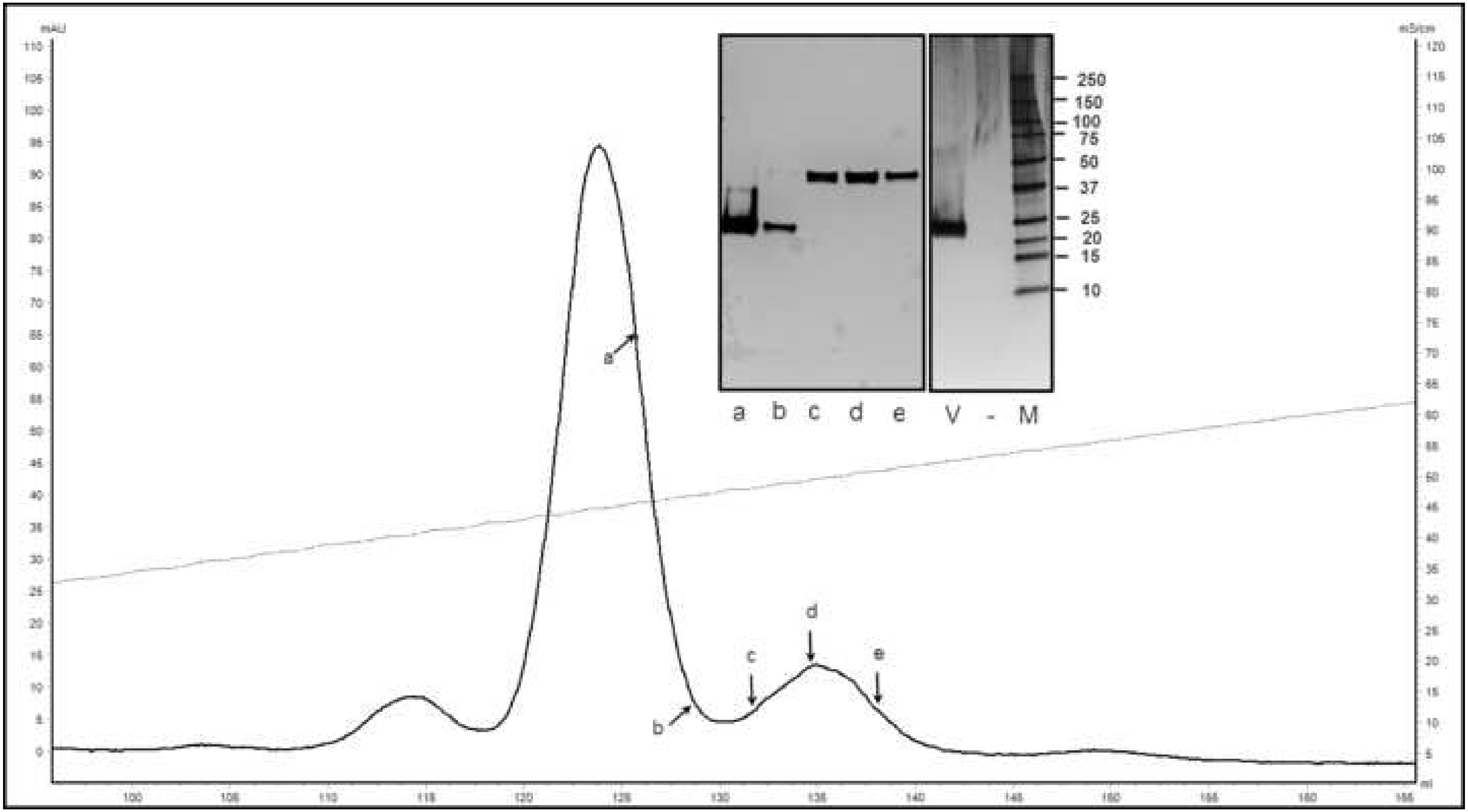

Figure3 Click here to download Figure Fig3.tif

(A)

```
        10         20         30         40         50         60
NLNRTNWPKK IFNIYWNVPT YFCHQHDVYF NELTKFDIKY NPKGNYRGDT ISLFYDPGNF
  *
        70         80         90        100        110        120
PAMVPLKNGT YDIRNEGVPQ KGNITVHLQQ FTKELDEIYP KKISGGIGVI NFNKWRPIFR

       130        140        150        160        170        180
RNVNNLKINK EVSIDLVRKE HPKWDKSMIE TEASNRFEKS ARIFMEKTLK LAKDIRNKNK

       190        200        210        220        230        240
WGYHGYPYCP TASTGNPSFD CDALAMNEND KLSWLFKYQD VLLPSVYVKH VLKPEEKIGL

       250        260        270        280        290        300
VRGSVKEAVR ISKKFEHLPK VLSYWWYAYE DKMDTFLTET DVKNTFREIL INGGDGIIIW

       310        320        330        340
GTMHDLNKEK CEKLKQYLST ILGPIAFKVM EAVKKRTPLN F
```

(B)

```
        10         20         30         40         50         60
SERPKRVFNI YWNVPTFMCH QYGLYFDEVT NFNIKHNSKD NFQGDKIAIF YDPGEFPALL

        70         80         90        100        110        120
PLKYGKYKIR NGGVPQEGNI TIHLQRFIEH LDKTYPNRNF SGIGVIDFER WRPIFRQNWG

       130        140        150        160        170        180
NMKIYKNFSI DLVRKEHPFW NKKMIELEAS KRFEKYARLF MEETLKLAKK TRKQADWGYY

       190        200        210        220        230        240
GYPYCFNMSP TNFVPDCDVT AMRENDEMSW LFNNQNVLLP SVYVRRELTP DQRIGLVQGR

       250        260        270        280        290        300
VKEAVRISNN LKHSPKVFSY WWYVYQDETN TFLTETDVKK TFQEIVINGG DGIIIWGSSS

       310        320        330
DVNSLSKCMR LREYLLTVLG PIAVNVTEAV N
```

Figure4 Click here to download Figure Fig4.tif

(A)

10 20 30 40 50 60
GLLPKCKLVP EQISFILSTR ENRNGVFLTL DSLKKGGILN KSDLSSTQVV FLIHGFISSA

70 80 90 100 110 120
NNSNYMDMTK ALLEKNDCMV ISIDWRNGAC TNEFQILKFI GYPKAVENTR TVGKYIADFS
*

130 140 150 160 170 180
KLLMQKYKVS LANIRLIGHS LGAQIAGFAG KEYQKFKLGK YPEIIGLDPA GPLFKSNDCS

190 200 210 220 230 240
QRICETDAHY VQIIHTSNNL GTERTLGTVD FYMNNGYNQP GCYYSFIGET CSHTRAVQYF

250 260 270 280 290 300
TECIRHECCL IGVPQSKNPQ PVSKCTRNEC VCVGLNAKRY PKTGSFYVPV ESKAPYCNNK

GKKI

(B)

10 20 30 40 50 60
NNYCKIKCRS GIHTLCKYGT STKPNCGRSV VKASGLTKAE KLEILKQHNE FRQKVARGLE

70 80 90 100 110 120
TRGNPGPQPP AKSMNTLVWN DELAQIAQVW ASQCKYGHDN CRNTAKYLVG QNIAEQSTTA

130 140 150 160 170 180
ASFEPVSNMV KMWSDEVKDY QYGSSKNKLN DVGHYTQMVW AKTKEIGCGN IKYIENGWHH

190 200
HYLVCNYGPA GNIGNEPIYE KK

Figure5 Click here to download Figure Fig5.tif

(A)

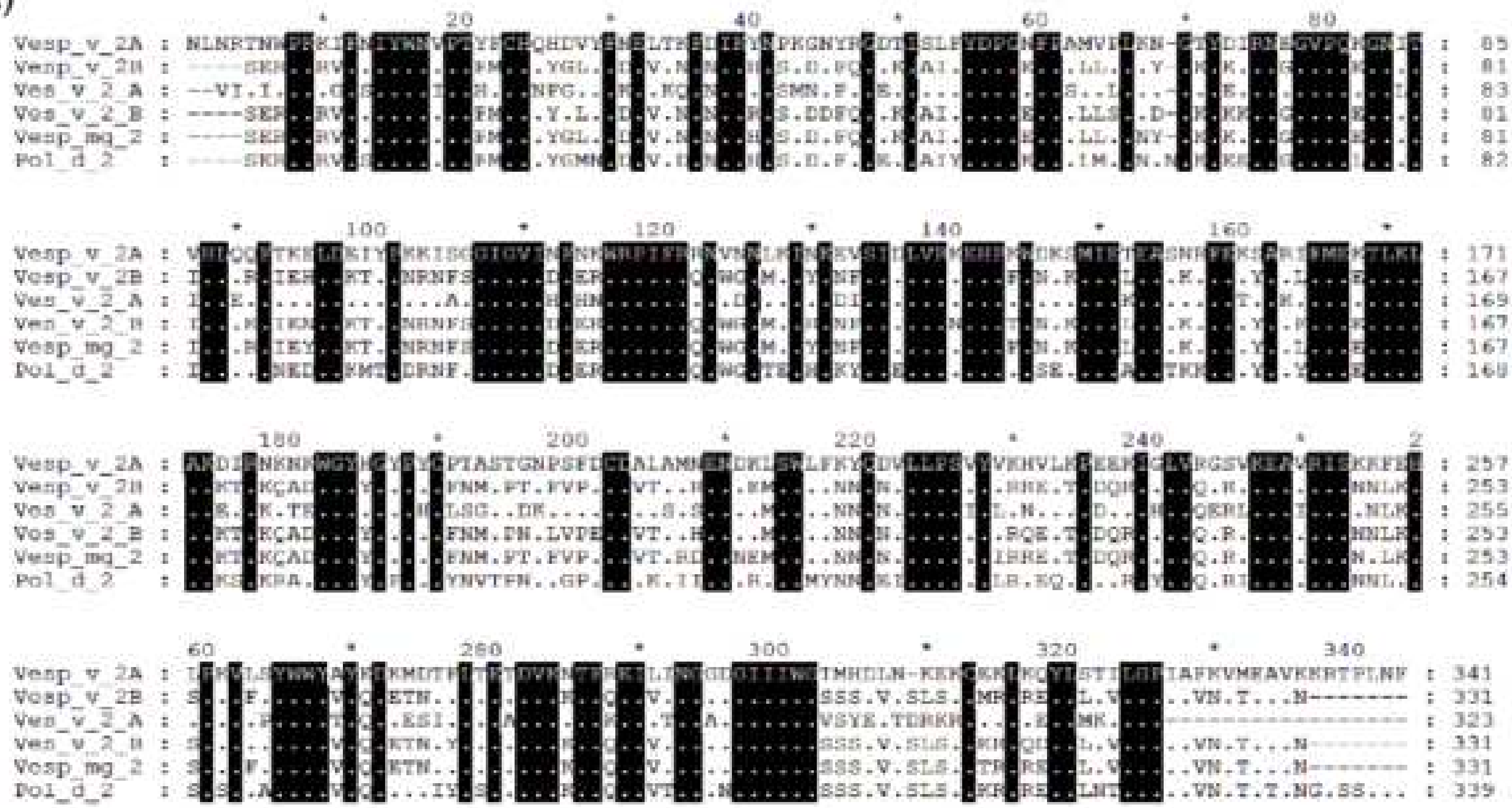

(B)

**Vesp v 2B (331aa)**	**vs Ves g 2**	**93%**
	**vs Vesp mg 2**	**97%**
	**vs Pol d 2**	**73%**
Vesp v 2A (323-341aa)	vs Ves g 2	69%
	vs "B" isoforms	58-60%
	Not present in *P.dominula*	--

Figure6

Click here to download Figure Fig6.tif

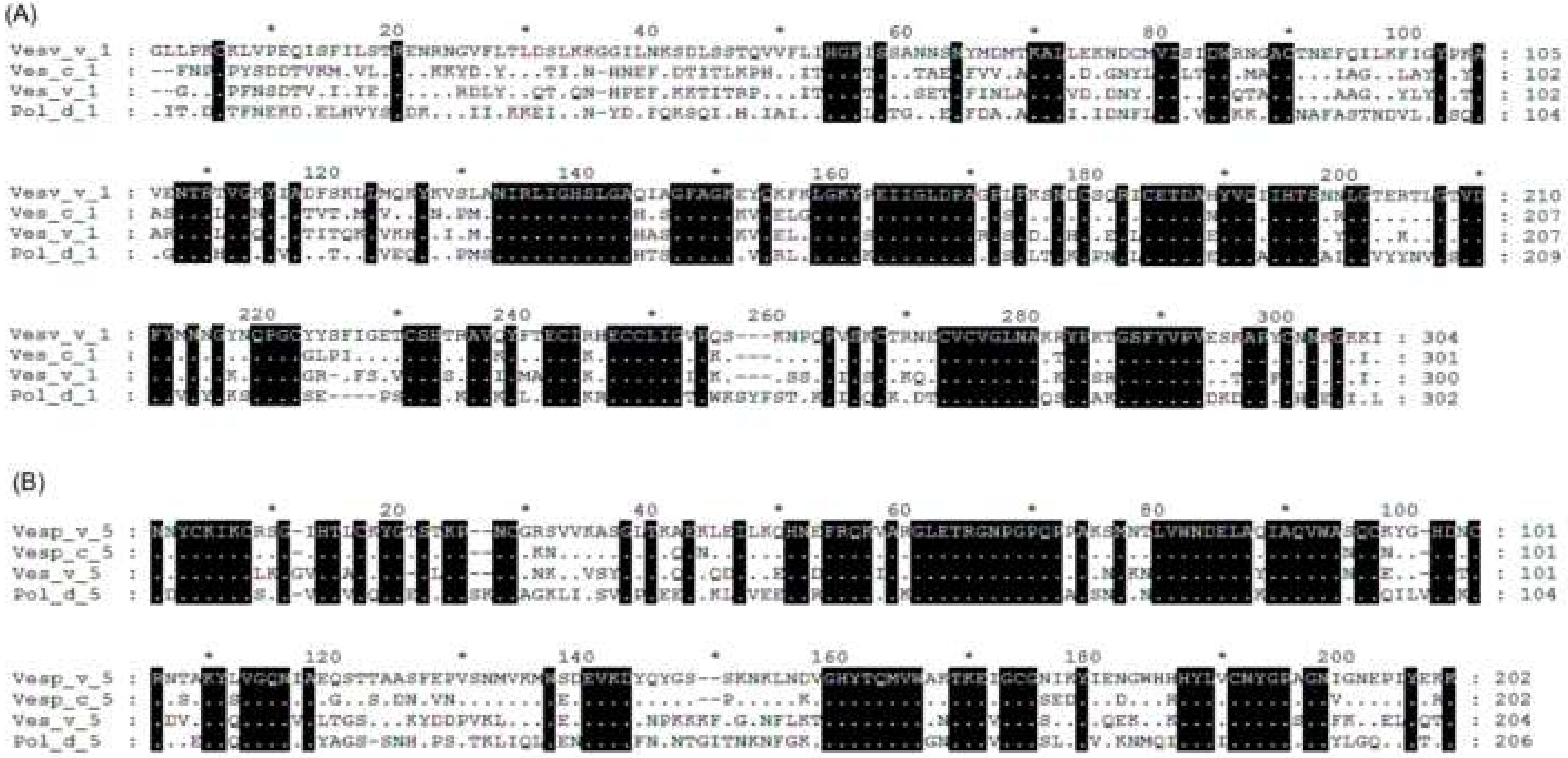

Supplement: Attachment — Submitted filename: PONE-D-19-22139_reviewer.pdf [file pone.0225672.s002.pdf]
