# Supplementary material for: Purification and molecular characterization of phospholipase, antigen 5 and hyaluronidases from the venom of the Asian hornet (Vespa velutina)
Source: PLoS One. 2020 Jan 10;15(1):e0225672. doi: 10.1371/journal.pone.0225672 (PMC6953831; doi:10.1371/journal.pone.0225672)
Supplement: Attachment — Submitted filename: Response to Reviewers.docx [file pone.0225672.s003.docx]

Dear Sir / Madam

This “Response to Reviewers” intends to rebut, answer and reply to the comments made by the Reviewers and the Academic Editors to the original manuscript.

In first place, several suggested corrections have been included in the manuscript, and the authors appreciate many of them, since they help to clarify and make more understandable the data presented. As example:

- Some extra data to be included in the Abstract, in order of being more informative;
- Some corrections to data repeated along the text (Abstract / Introduction /Conclusion sessions) or that were incorrectly expressed; e.g. line 37, info in lines 58-59, non-relevant info in Fig.3, text in lines 249-254, not using the word “several” when referring to the 4 components isolated, etc.etc.
- Besides, in further reading of the manuscript text, other typewriting errors have been included (they all can be seen in the version with “track changes”). E.g. the term Hymenoptera has been corrected throughout the text.

In second place, please consider that we have confirmed our “Data Availability Statements”: we have deposited the data from our experimental determinations, so that as soon as the manuscript is accepted they will be publicly available, including the whole set of data for the Mass Spectrometry analyses of the purified proteins (included in the MassIVE server), as well as making publicly available the sequences determined in the UNIPROT databases and the WHO/IUIS Allergen databases.

- With respect to the MassIVE platform, the ProteomeExchange identifier, is PXD015381, and this has been stated in the Title page of the manuscript, and again in the Materials and Method and in the Results (in Figure 4’s legend) sections.

IN RESPONSE TO REVIEWER #1

We have considered the comments from the reviewer concerning the inclusion in the Abstract of some of the relevant data included in the manuscript. We have modified the Abstract accordingly, referring to these comments:

*Highlights should be pointed out by the authors in the Abstract emphasizing the contribution of this study.*

*The Abstract could be much more informative regarding the specific results obtained for the wasp venom components studied in comparison to the literature data.*

*Consider use this specific result as part of the Abstract (referring to the text in which we wrote that “* An interesting feature for Vesp v 1, is that it is a glycosylated phospholipase…“)

*In my opinion part of these specific results should be used in the Abstract in place of the very general phrase "The overall data confirm the high similarity of the V.velutina components with the analogous components of other Vespa and Vespula species." (in particular, when we wrote about the striking similarities among hyaluronidases of Vespa and Vespula).*

OTHER SPECIFIC COMMENTS FROM THE EDITOR/REVIEWERS

1) With regard to Figure Legends (Figure 1, 2 and 5)

“*Please, consider to use symbols like "(____) and (----) in legends, instead of sentences to explain differences among the line formats inside graphics*.”

- Those Figures have been improved in quality, also adding colour to the different lines drawn, and therefore the explanations in the corresponding Figure legends where simplified accordingly.

Figure 2 legend (and the corresponding text in the Results section) had these 3 comments:

“*Consider to proceed like indicated in legend of Fig 1*.”

“*As the blank lane seems to have some sample, I guess it would be suitable explain what happened in this case or to which kind of sample it is correspondent”*

*“It would be interesting if the levels of hyaluronidase activity were also demonstrated in the chromatographic profile.”*

As stated before, symbols have been used in the Figure legend

Besides, the SDSPAGE shown in the right panel, has been repeated and the result presented in the new Fig 2; the same sample has been applied (0.25µg of Vesp v 5), and a correct silver stain is now being shown (the previous SDSPAGE was just excessively stained, and the SDSPAGE showed artefacts in the “blank” lane which did not contain anything at all; moreover, the MW markers had not run correctly in the SDSPAGE). Therefore, we decided to repeat the experiment, and show that the excellent purity of Vesp v 5 is just the same (as stated in the text this was already demonstrated also by the N-terminal analysis).

Finally, as suggested by the Reviewer, we have also included in Figure 2 the “relative activity” for hyaluronidase enzymatic activity (including explanations both in the Figure legend and also in the text):

Please note that this is just a “relative” and not a precise quantitative measure (as it was mentioned before). We only “followed” some fractions with activity, in order to locate the presence of Hyaluronidases by this technique. We had not the intention of precisely quantifying this activity, and therefore, in the agarose-activity plate, we only used two dilutions from the “positive control” (the CBER reference, which is a solution of 1mg/ml crude venom extract), and therefore we cannot transform the data quantitatively from this experiment.

Therefore, we just show were we could observe clear positive activity (***) and slightly less activity (*), also showing the cm of the wheal that we measured in the agarose plates.

Please, consider that all the figures sent with this manuscript have been previously sent to the “server” that PLOS suggested (the PACE digital diagnostic tool), so that they comply with the requirements from the Journal. Besides, considering the comments from one of the Reviewers, the quality of the graphics and the SDSPAGE presented were improved as much as possible (see the specific reply to REVIEWER #2 below).

REPLY TO REVIEWER#2’s COMMENTS

This manuscript just describes the molecular characterization and purification of a phospholipase (Vesp v 1), an antigen 5 (Vesp v 5) and two hyaluronidase isoforms (Vesp v 2A and Vesp v 2B) from the Vespa velutina nigrithorax wasp venom (Hymenoptera, Vespidae). This wasp is known as yellow-legged Asian Hornet. It Is a species of hornet indigenous to Southeastern Asia. It is of concern as an invasive species in some other countries and in Portugal, it predates honey-bees and other insects. It has been studied for causing major human accidents in the North-Western parts of the Spanish coast.
In case this paper is approved for publication by PlosOne, I would rather suggest the authors to answer the following questions:
- What is effectively the extent of this wasp on human accidents?

The extension of this wasp has been of social concern, mostly in the North West part of Spain since 2016, when it’s expansion has been remarkable. Not many scientifically published data, but the “Regional Governments” are taking care of the social problem (also affecting beekepers), helping people to be aware of the characteristics of the invasive wasp, more active than the autochthonous *Vespa crabro*, and with many nests containing numerous individuals (up to around 10,000 hornets working and “defending” their nests).

Please, consider the official information provided to the public since 2018 by this institution (sorry for the Spanish or the “Galician” in which is written):

- Data taken from <https://mediorural.xunta.gal/es/areas/ganaderia/apicultura/vespa_velutina/>
- Evolution of the nests found in this area (the dark red shows the areas with more nests):

In all this area of Spain (and North of Portugal), people live very much in small villages; they are close to the nests, and an Emergency Service has been organized (phone number 112) for trying to “eliminate” nests nearby their country-houses.

- It is quite frequent that they get stung, and if previously sensitized to this or similar wasps, they can suffer anaphylactic shocks (3 fatal cases in the summer of 2018, 2 fatal cases so far in 2019, including one in the nearby Asturias region). Most of the people were just “cleaning their garden or the surrounding of their country house)
  - Please consider that not always people are allergic to *V.velutina*, and not in all the “fatal cases” reported the Asian hornet was the causative insect. But the cross-reactivity is high, and people allergic to Vespula, could have hypersensitivity also to the Asian hornet.
  - July 2018 newspaper *article* on the 3 fatal cases (<https://www.lavozdegalicia.es/noticia/galicia/2018/07/16/tres-muertos-velutina-galicia-realizaban-trabajos-fincas/0003_201807G16P4991.htm>)
  - June 2019 newspaper article on the case in Asturisa (<https://www.elmundo.es/cronica/2019/06/07/5cf9653021efa0b24e8b46b0.html>)

Also the Hospitals and medical services of the region have been very busy with the alarm that this invasion and the presence of this hornet is causing.

Until know, an *in vitro* IgE test, specific for V.velutina was not available. Now, it has been provided by us, as Research collaboration to several medical groups (and also the purified components will be available). This is what the Spanish National Allergic Society (SEAIC) is informing about the absence of this test (sorry, in Spanish):

(January 2016 information, taken from <https://www.seaic.org/profesionales/blogs/alergia-a-himenopteros/comunicado-del-cah-sobre-vespa-velutina-2.html>)

- What is the significance of the translational medicine standpoint for this type of research, I´d say, to mitigate allergic reactions (treatment) and better diagnosis?

As also indicated by the Spanish Allergology Society (SEAIC), and by some previously published data (mentioned in the manuscript), the available extracts (in particular if they are prepared from the genus *Vespula*) could protect these patients by immunotherapy. Our data support the similarity Vespa/Vespula; the complete sequences data are new and are being made available to the Scientific community, and our PURIFIED REAGENTS will be available for a better diagnosis of the patients (the diagnosis step is of utmost importance for deciding how to treat the patients, as we published in 2012, together with several groups of the Hymenoptera Committee of the SEAIC, also isolating some of these components from *Vespula* and *Polistes* and using them in Component Resolved Diagnosis). More precise data will be available soon, hopefully, when the medical doctors can test their patients with the different components and evaluate their treatments.

- How can differences in sensitization to immune reaction between Vespula and Polistes be explained since they are very close phylogenetically (daughter group), since both belong to the Vespidae family?

As we published in 2012 (see references in Allergy 67:528–536 and Allergy 67:966–969), and as other authors had published before (Severino et al. (2006) Allergy 61: 860-863) the greatest problem is a correct diagnosis. These studies showed that the Vespula/Polistes differentiation was very important, and the correct diagnosis and specific treatment of patients was more effective. Our company (after our data gave such clear evidence) decided to provide patients in Europe, since June 2006, with a preparation from the European species *Polistes dominula*, instead of the “American mix” that was used before. Many people could be have been wrongly diagnosed, before (as shown by Severino et al.)

In spite of the overall similarity in their allergenic components, using the wrong extract for immunotherapy, can lead to a patient incorrectly protected, and there are fatal cases (or cases in which the treatment was changed and the correctly-treated-patient protected):

- Bonadonna P et al (2007) Allergy 62:966-7 (Treatment with American Polistes venom was ineffective in an Italian patient allergic to European Polistes).
- Dalmau G et al (2012) JIACI 22(2):135-6. (Importance of Controlled Sting Challenge and Component-Resolved Diagnosis in the Success of Venom Immunotherapy)

Probably, the problem is that *Polistes* is present and gradually extending its presence mostly in Southern European countries. In Germany and Northern European countries, the presence of *Polistes* is scarce, and they are still not worried about it. But *Polistes* is also “extending” to other regions in Europe, as it has done in the U.S. (we mentioned this, in our 2012 publication)

- The most important concern of this manuscript is: authors used a pool of wasp venoms from all over Europe. Doesn´t this make it difficult because of individual variations in wasp venoms from different regions of Europe? I mean, antigen detection by antibodies should be specific as well as treatment. We must know exactly which immunogenic compounds are present in a venom gland of a specific species for the detection and later medication. Therefore, if you use a pool, how effective can these two (diagnosis and treatment) factors be, in addition to those molecules evidenced in this study? Is there any study showing that there are differences between venoms of this wasp, due to food, from different regions?

The venoms used in “allergic immunotherapy” (and also in diagnosis) are generally directly extracted from the venom sac of previously identified wasps. These venoms (from wasps and honeybees) are a very efficient “reagent” for treating this type of hypersensitivity: its effectiveness is above 90%, and patients are considered to be “cured” of this life-threatening disorder after 3-5 years of treatment (this protection is long-lasting, except for patients with Mastocytosis, who need to be vaccinated during all their life).

Please, consider these recent reviews/publications, one from the USA and the other from European scientist for additional information on what I have stated on the use of venoms for treatment and their efficacy in allergic patients:

- Oppenheimer J and Golden DBK (2018) Annals of Allergy, Asthma Immunology, doi: 10.1016/j.anai.2018.06.006. (Hymenoptera Venom Immunotherapy: Past, Present, and Future)
- Schiener M. et al (2017) HUMAN VACCINES & IMMUNOTHERAPEUTICS 13(10):2467–2481, <https://doi.org/10.1080/21645515.2017.1334745>. (Allergen-specific immunotherapy of Hymenoptera venom allergy – also a matter of diagnosis)

Indeed, the immunogenic compounds extracted from a venom gland, can vary due to different situations in the wasps from which de venom is extracted, as the Reviewer mentions. But I would like to mention the following:

- The venom we have used is obtained from ALK Source Materials Inc., USA, formerly known as “Vespa Laboratories”, a company with very long experience in collecting venom from diverse Hymenoptera.
- The collection of the venom is always performed in the same way: careful identification of the species (in the case of wasps and hornets), capture by aspiration (with a specifically prepared vacuum-machine) and immediate freezing of the insects (so that they are not stressed and the venom sac content does not suffer possible variations), and finally, the venom is extracted individually at the lab facilities of the mentioned company, by thawing a controlled number of individuals, extracting the venoms sac by pulling the sting, and homogenizing the venom extract in adequate buffers (then, the venom is controlled by means of total protein, protein profile, and enzymatic activity of the proteins present in the extract along the whole process until they reach the medical doctors; this process is GMP-controlled, and approved by the FDA)
  - I.E. the extract is very much controlled and produced in a way that little variations should be expected from batch to batch, and variation in the main allergenic components would be detected during the process
  - This is the way “venoms preparation” have been prepared and used for diagnosis and immunotherapy, and this is the source material that we have used for purifying the components of *V.velutina*, and also from *Vespula* and *Polistes* species in the past (Monsalve et al.2012; Allergy 67:528–536)

Therefore, it is true it’s a “pool of venoms” from individual insects collected in Europe what is being used for treating patients and also for the work we have performed (same source material). In addition, I want to stress on the importance of the results we have found (as I mentioned in the manuscript), regarding the sequence findings we present (mainly the precise data that the MS/MS study provides):

- The sequences of the natural proteins we have isolated, match with no differences at all with the transcriptomic data that were obtained before 2015 in *Vespa velutina* nigrithorax species that were collected in China (Liu et al 2015 Nature Scientific Reports 5(9454) DOI: 10.1038/srep09454, reference cited in the text).
- We looked for other isoforms of Vesp v 5 or Vesp v 1, and they were not found (as mentioned in the manuscript, two isoforms for Vesp v 2 were found); i.e. the only sequences found in the transcriptomic data from the RNA of Chinese species, are coincident with the natural proteins purified from venom of specimens that have invaded Europe more than 10 years ago. I am not aware of other publications showing this, or differences in the venoms of wasps due to food or other geographical issues.
  - Taking into account that we consider that these data are quite relevant, and also considering a comment from this Reviewer that the Discussion is scarce, we have added more comments regarding these specific issues mentioned in this reply. Hope the Reviewer and the editors of PLOS ONE, also consider that these facts are relevant.

Therefore, the “stability” of the venom extract preparation, and the controlled procedure that is constantly being considered, is a warranty that the “reagents” used for treating patients are stable and contains the components that actually cause the anaphylactic shock on the allergic patients to be treated.

- Is there any specificity and / or similarity between the isolated molecules and those already well-known from other sources of hymenoptera and / or other animals?

The similarities among different Hymenoptera components are discussed in the manuscript, after the multiple sequence alignments performed. These comparisons have been circumvented to the closest species existing as clinically relevant as potential allergenic components, and the similarities with other homologous proteins in animals have not been considered as relevant.

The specificity as IgE-detecting reagents is to be tested with the sera of allergic patients, and we think it will be interesting. But, as said when we sent the manuscript for publication, we expect that these data will be provided my the medical doctors, with their patients and with their knowledge, so that the conclusions they reach, on the usefulness of the reagents we will be providing, and on the correct treatment of patients, will be their responsibility (there are many different groups involved in the North-West part of Spain, and each of them have to provide this experience and their conclusion, without the bias that could be foreseen considering we are a Research and Development department of a private company). I think we have provided new data and new tools for a deeper knowledge on these venom components, and hopefully these reagents will be useful (we have no intention at all to commercialize these as diagnostic tools; we have provided the reagents for research purpose).

- Actually, the discussion of the manuscript is contained from page 335 to 362. Therefore, it is quite powerless and does not, in my view, match the level of the journal.

We tried to present a "Result and Discussion" session starting in line 145, and tried to discuss the results while presenting them. Maybe we did it in a concise way, trying to enhance the most relevant results. Nevertheless, considering this Reviewer’s specific comments, as mentioned before, more discussion has been added to this section, including some data that are indeed relevant in our opinion. We hope that mentioning some relevant issues, when describing the result, and then again while discussing other issues does not make that these comments are redundant.

Indeed, our data are more related with the "new molecular data" that we have obtained from this characterization, make these data available to the scientific community, and suggest how they could be used (they will be available in the UNIPROT database as soon as the manuscript is ready for publication, as it is the case for the data available at [www.allergen.com](http://www.allergen.com), who have recently accepted our proposed nomenclature for the *Vespa velutina* allergens). Again, we expect the purified components are now used as "specific reagents" for a better diagnosis and understanding of the allergic reactions to these Hymenoptera, and more data will be available in the future.

- Finally, there is no consistent discussion of isolated molecules and effective actions to solve medical and immunological problems.

I must insist that we are not presenting data with sera of patients, since the sera belong to different clinical groups, and they will have to analyze the data with the reagents we will provide (and with the knowledge we intend to make public if the data are published), and the medical groups should really confirm on the usefulness of the approach for correctly treating these patients.

With the data available so far, we could only “speculate” on the similarities between Vespa/Vespula/Polistes, which is something we suggest, and we think that the higher similarities between *Vespa* and *Vespula* suggest that the latter is the closest venom extract to that of the Asian hornet, for correctly treating the patients (considering that nowadays, only *Vespula* or *Polistes* extracts are commercially available in Spain for treatment of wasp allergy). But the real data and conclusions to solve medical and immunological problems are still to come.

From our side, we think the “contribution” with the new data presented is really remarkable, as well as providing the purified proteins for performing specific IgE tests *in vitro*. I hope the Reviewer is a bit more convinced on our arguments, due to the relevance this Asian hornet is acquiring in specific parts of North-West Spain.

- Which chromatography method was used: FPLC or HPLC?

FPLC on conventional liquid chromatography has been used. The purification method follows what it had been published already, and the columns used, and mentioned in the text, are all to be considered FPLC columns:

- PC-Sepharose / Mono-S column / Heparin Sepharose / and size-exclusion chromatography (SEC) in Superdex75
  - It is true the latter had not been specifically mentioned in the first draft of the manuscript, but with the suggested corrections this chromatography is now mentioned)

- What is a "direct analysis from the protein in solution"?

This sentence tried to express that the sample for N-terminal-sequencing analysis is analyzed from an aqueous sample after its purification (in this way, this analysis is also providing data on the purity of the sample), IN CONTRAST with what is explained in the following paragraph for the sample analyzed by nLC-MS/MS (nano Liquid Chromatography tandem mass spectrometry) in which the “analyses were performed from the bands extracted from an SDSPAGE stained with Coomassie blue”. This “n LC MS/MS” analysis requires this procedure to avoid buffer components interfering in the very sensitive analysis to be performed.

- No reagents used in the study have the company's city and country of origin.

This issue has been addressed throughout the manuscript, including the data suggested (except for the reagents used at the Mass Spectrometry Service (for the nano Liquid Chromatography tandem mass spectrometry analysis, since their protocol uses acetonitrile / TFA, etc., but do not mention the provider of such reagents).

- The figures are not of good quality and need to be improved.

Quality of the figures has been cautiously improved.

- In particular, the chromatographic profiles of Fig 1 and Fig 2 have been now represented with a better software (GraphPad), instead of the elution profiles that the UNICORN software could provide (the data are exported from UNICORN and represented in the new Figures, taking care of showing the data so that they are clearly visible, including their axes legends and the values represented).
- Besides, as mentioned before in this document, the result showing the purity of Vesp v 5 (right panel of inset of Fig 2) has been prepared again (as specified before, the experiment has been repeated with the same sample, and the technical artefacts observed in the silver-stained SDSPAGE of the first draft have now been solved, since it was just a matter of extra-silver staining, and not-correct migrations of the MW markers).
- Finally, consider that all the figures sent together with this manuscript have now been sent to the “server” that PLOS suggested (the PACE digital diagnostic tool), previously to the sending of this revised manuscript, so that they comply with the requirements from the Journal.

We hope now the quality is adequate for publication.

With kind regards

Rafael Monsalve, PhD
